# Use of serotonin reuptake inhibitors and risk of subsequent bone loss in a nationwide population-based cohort study

**DOI:** 10.1038/s41598-021-92821-9

**Published:** 2021-06-29

**Authors:** Sunyoung Kang, Minkyung Han, Chun Il Park, Inkyung Jung, Eun Hwa Kim, Young Jun Boo, Jee In Kang, Se Joo Kim

**Affiliations:** 1 Institute of Behavioral Science in Medicine, Yonsei University College of Medicine, Seoul, Republic of Korea; 2 Biostatistics Collaboration Unit, Department of Biomedical Systems Informatics, Yonsei University College of Medicine, Seoul, Republic of Korea; 3 Department of Psychiatry, CHA Bundang Medical Center, CHA University, Seongnam, Republic of Korea; 4 Division of Biostatistics, Department of Biomedical Systems Informatics, Yonsei University College of Medicine, Seoul, Republic of Korea; 5 Department of Psychiatry, Yonsei University College of Medicine, Yonsei-ro 50-1, Seodaemun-gu, Seoul, 03722 Republic of Korea

**Keywords:** Endocrinology, Psychiatric disorders

## Abstract

This study examined whether the use of SRIs is associated with an increased risk of bone loss using a nested case–control design with a nationwide population–based cohort in Korea. Using the Korean National Health Screening Cohort, subjects newly diagnosed with osteoporosis or osteopenia (n = 55,799) were matched with controls (n = 278,995) at a ratio of 1:5. We stratified the participants by their time-dependent use of SRIs and sex and controlled for various confounders, including lifestyle habits, laboratory data, and comorbidities. Conditional logistic regression showed that both recent and former users of SRIs had an increased risk of subsequent bone loss compared with non-users: men [recent users: odds ratio (OR) 1.35, 95% confidential interval (CI) 1.20, 1.53; former-users: OR 1.10, 95% CI 1.01, 1.20]; women (recent users: OR 1.38, 95% CI 1.28–1.48; former-users: OR 1.07, 95% CI 1.02, 1.21). The use of SRIs was associated with an increased risk of bone loss in both men and women. In particular, the association was stronger in recent users. These findings provide population-level evidence for the risk of bone loss associated with SRI exposure and highlight the importance of monitoring the bone health of SRI users.

## Introduction

The use of antidepressants, especially serotonin reuptake inhibitors (SRIs), has become increasingly common in recent decades all around the world^1–5^. Reports of low bone mineral density (BMD)^6–8^ and bone fractures^9–19^ in people taking antidepressants have raised the concern that SRIs could have adverse effects on bone. A meta-analysis of 16 studies reported that users of selective serotonin reuptake inhibitors (SSRIs) are 1.61 times more likely to develop bone fractures than non-users (Relative risk 1.61 95% CI 1.49, 1.74)^15^. In addition, some studies found that the use of serotonin-norepinephrine reuptake inhibitors (SNRIs) can be associated with an increased risk of bone fractures^16,17^. Although those fracture-risk findings suggest a possible link between use of SRIs and bone health, any real connection between them remains unclear^20^, and the risk of bone fractures among people taking antidepressants could be related to other factors, such as falls and depressive status.

Findings about the adverse effects of SRI use on BMD are limited and mixed. Although several studies reported that a history of taking SSRIs predicted decreased BMD in both men and women^6–8,18^, some studies found no significant association between them^21,22^. A meta‐analysis of 4 studies examining the association between antidepressants and BMD in women showed that antidepressant was not associated with lower or higher BMD^23^. A cohort study (173 men and 323 women) reported that taking antidepressants was associated with low BMD in women (Coefficient -0.141, 95% CI -0.263, -0.020) but not in men (Coefficient 0.073, 95% CI -0.086, 0.232)^22^. Moreover, current users of antidepressants were reported to show a significantly higher risk of osteoporotic fractures than former users^17^. These inconsistent results across studies might be attributable to small sample sizes, different study designs, sample characteristics such as sex differences, different duration of antidepressant use, and sampling bias. More research into the independent effect of SRIs on bone health in a large unbiased clinical sample controlled for various confounders is needed to improve the safety of SRI treatment and the healthcare quality.

In this study, we used a nationwide population-based Korean cohort to examine whether the use of SRIs is associated with subsequent bone loss. We used a nested case–control design with a 1:5 matching ratio of cases to controls and stratified the participants according to their time-dependent use of SRIs and their sex. In addition, we controlled for various potential confounders such as laboratory test results and lifestyle factors that could affect bone health.

## Results

**Figure 1:**
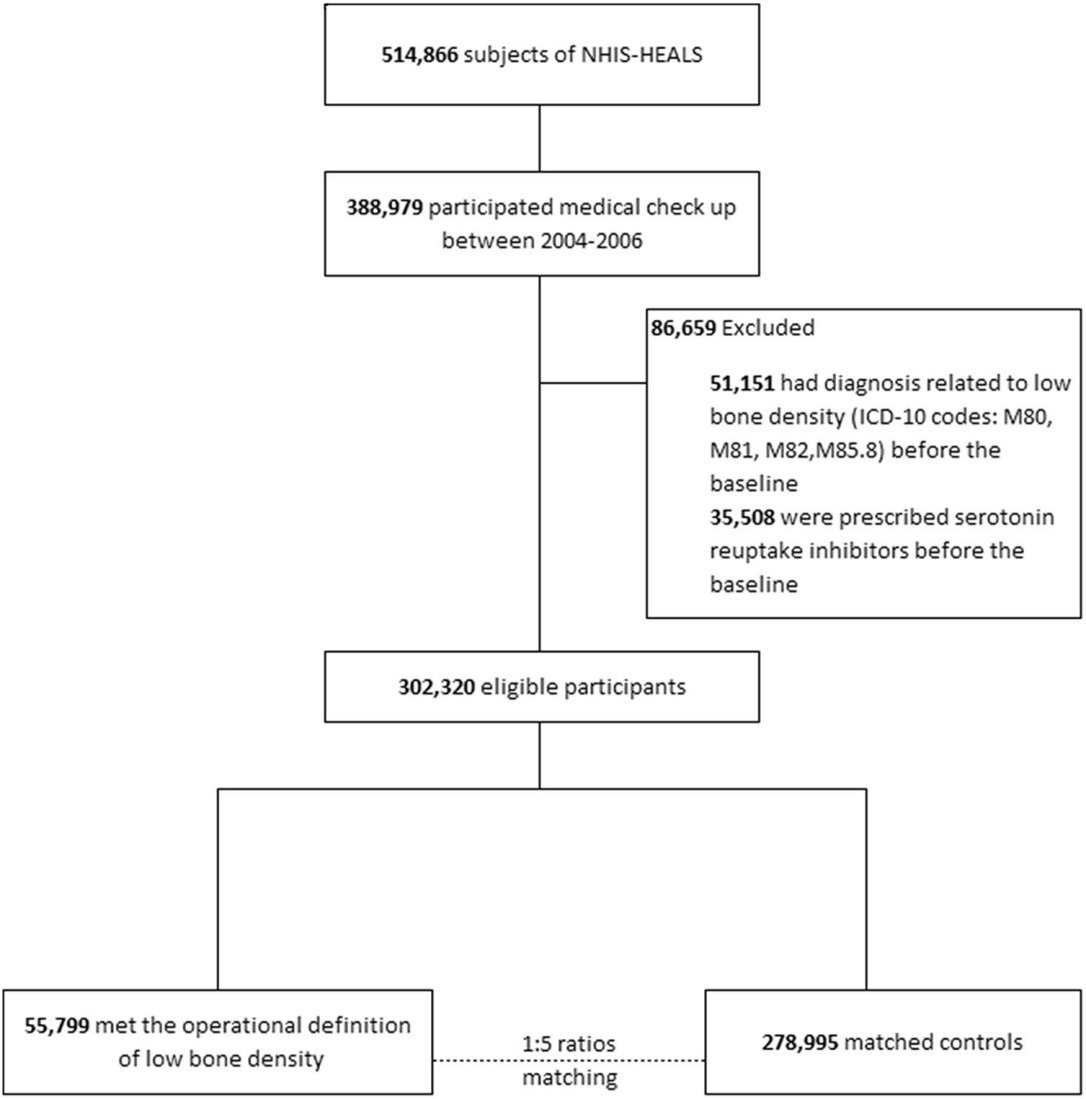
Flow chart of the study process.

We identified 388,979 individuals who had received a medical check-up between Jan 2004 and Dec 2006. We excluded participants with a previous diagnosis related to bone loss (n = 51,151) or exposure to SRIs during the wash out period (n = 35,508). Using a 1:5 matching ratio of cases to controls, controls without any diagnosis related to bone loss (n = 278,995) were randomly selected for each case in the group with low bone mass [n = 55,799; 43,313 Women (77.6%); mean (SD) age, 58.0 (9.4) years]. A detailed flow chart is provided in Fig. 1.

The baseline characteristics of the study sample are presented in Table 1. The rate of participants who never used SRIs was lower in subjects with a new diagnosis of low bone density than in those without such a diagnosis. At the same time, subjects in the case group were more likely to be classified as former or recent users of SRIs than those in the control group. In both sexes, subjects in the case group were more likely than the controls to take glucocorticoids, GnRH agonists, anticonvulsants, thiazolidinediones, antipsychotics, benzodiazepine, and TCA. Men and women who were newly diagnosed with low bone density had a higher prevalence than controls of using androgen deprivation therapy and aromatase inhibitors, respectively. Subjects with low bone density were more likely to take DMPA and estrogen or a combination of estrogen and progesterone, but those outcomes occurred only in women. The low bone density group was also more likely than the controls to report high CCI, low systolic and diastolic blood pressures, low fasting blood glucose level, and low total cholesterol level; and perform little exercise. The case group presented with lower hemoglobin levels and higher AST than the controls, but this outcome occurred only in men. Participants with osteoporosis had lower urine protein and ALT levels, and those results were driven by women.

**Table 1:** Baseline characteristics of the study population.

Characteristic	Total			Men			Women		
	Low bone density	Normal	P-value	Low bone density	Normal	P-value	Low bone density	Normal	P-value
	(n = 55,799)^a^	(n = 278,995)^a^		(n = 12,486)	(n = 62,430)		(n = 43,313)	(n = 216,565)	
**Age, mean ± SD**	58.0 ± 9.4	58.0 ± 9.4		61.0 ± 9.6	61.0 ± 9.6		57.1 ± 9.1	57.1 ± 9.1	
**Serotonin reuptake inhibitors use**			< 0.001			< 0.001			< 0.001
Never used, no. (%)	51,186 (91.7)	263,413 (94.4)		11,296 (90.5)	58,777 (94.2)		39,890 (92.1)	204,636 (94.5)	
Former user, no. (%)	3151 (5.7)	11,358 (4.1)		755 (6.0)	2574 (4.1)		2396 (5.5)	8784 (4.1)	
Recent user, no. (%)	1462 (2.6)	4224 (1.5)		435 (3.5)	1079 (1.7)		1027 (2.4)	3145 (1.4)	
**Medication**^**d**^									
Glucocorticoids, no. (%)	46,024 (82.5)	203,843 (73.1)	< 0.001	10,610 (85.0)	47,318 (75.8)	< 0.001	35,414 (81.8)	156,525 (72.3)	< 0.001
Aromatase inhibitors, no. (%)^b^	225 (0.4)	199 (0.1)	< 0.001				225 (0.5)	199 (0.1)	< 0.001
Androgen deprivation therapy, no. (%)^b^	126 (0.2)	278 (0.1)	< 0.001	126 (1.0)	278 (0.5)	< 0.001			
GnRH agonists, no. (%)	201 (0.4)	402 (0.1)	< 0.001	134 (1.1)	266 (0.4)	< 0.001	67 (0.2)	136 (0.1)	< 0.001
Anticonvulsants, no. (%)	3819 (6.8)	13,355 (4.8)	< 0.001	1129 (9.0)	3540 (5.7)	< 0.001	2690 (6.2)	9815 (4.5)	< 0.001
Thiazolidinediones, no. (%)	1072 (1.9)	4625 (1.7)	< 0.001	386 (3.1)	1638 (2.6)	0.004	686 (1.6)	2987 (1.4)	0.001
DMPA, no. (%)^c^	794 (1.4)	2739 (1.0)	< 0.001	1 (0.0)	0 (0.0)	0.167	793 (1.8)	2739 (1.3)	< 0.001
Antipsychotics, no. (%)	2184 (3.9)	8231 (3.0)	< 0.001	671 (5.4)	2089 (3.4)	< 0.001	1513 (3.5)	6142 (2.8)	< 0.001
Benzodiazepine, no. (%)	43,877 (78.6)	186,829 (67.0)	< 0.001	9888 (79.2)	41,627 (66.7)	< 0.001	33,989 (78.5)	145,202 (67.1)	< 0.001
TCA^c^, no. (%)	10,855 (19.5)	35,226 (12.6)	< 0.001	2658 (21.3)	8128 (13.0)	< 0.001	8197 (18.9)	27,098 (12.5)	< 0.001
Estrogen or estrogen + progesterone combination, no. (%)	6175 (11.1)	18,678 (6.7)	< 0.001	5 (0.0)	27 (0.0)	1	6170 (14.3)	18,651 (8.6)	< 0.001
**Household income**^**e**^			< 0.001			< 0.001			< 0.001
Low, no. (%)	11,053 (19.8)	54,479 (19.5)		1978 (15.8)	9897 (15.8)		9075 (20.9)	44,582 (20.6)	
Mid-low, no. (%)	9418 (16.9)	45,356 (16.3)		1857 (14.9)	8312 (13.3)		7561 (17.5)	37,044 (17.1)	
Middle, no. (%)	8738 (15.7)	42,469 (15.2)		1879 (15.0)	9118 (14.6)		6859 (15.8)	33,351 (15.4)	
Mid-high, no. (%)	10,518 (18.9)	52,449 (18.8)		2582 (20.7)	12,819 (20.5)		7936 (18.3)	39,630 (18.3)	
High, no. (%)	16,072 (28.8)	84,242 (30.2)		4190 (33.6)	22,284 (35.7)		11,882 (27.4)	61,958 (28.6)	
**CCI**^c,f^ **(past two years)**	1.2 ± 1.4	1.0 ± 1.3	< 0.001	1.4 ± 1.6	1.1 ± 1.4	< 0.001	1.2 ± 1.4	0.9 ± 1.3	< 0.001
**BMI**^**c,e**^			< 0.001			< 0.001			< 0.001
< 18.5, no. (%)	1720 (3.1)	6238 (2.2)		552 (4.4)	1716 (2.7)		1168 (2.7)	4522 (2.1)	
18.5–22.9, no. (%)	22,510 (40.3)	100,407 (36.0)		5345 (42.8)	22,459 (36.0)		17,165 (39.6)	77,948 (36.0)	
23.0–24.9, no. (%)	14,577 (26.1)	75,222 (27.0)		3266 (26.2)	18,030 (28.9)		11,311 (26.1)	57,192 (26.4)	
≥ 25.0, no. (%)	16,965 (30.4)	97,028 (34.8)		3322 (26.6)	20,207 (32.4)		13,643 (31.5)	76,821 (35.5)	
Missing, no. (%)	27(0.1)	100(0.0)		1(0.0)	18(0.0)		26(0.1)	82(0.0)	
**SBP/DBP**^**c,e**^			< 0.001			< 0.001			< 0.001
SBP < 130/DBP < 85 mmHg, No. (%)	29,112 (52.2)	136,091 (48.8)		5497 (44.0)	25,200 (40.4)		23,615 (54.5)	110,891 (51.2)	
SBP ≥ 130/DBP ≥ 85 mmHg, No. (%)	26,663 (47.8)	142,808 (51.2)		6986 (56.0)	37,213 (59.6)		19,677 (45.4)	105,595 (48.8)	
Missing, no. (%)	24 (0.0)	96 (0.0)		3 (0.0)	17 (0.0)		21 (0.1)	79 (0.0)	
**Fasting blood glucose**^**e**^, **mean ± SD**			< 0.001			< 0.001			< 0.001
< 100 mg/dl	39,478 (70.8)	192,012 (68.8)		8025 (64.3)	38,335 (61.4)		31,453 (72.6)	153,677 (71.0)	
≥ 100 mg/dl	16,264 (29.2)	86,729 (31.1)		4448 (35.6)	24,045 (38.5)		11,816 (27.3)	62,684 (28.9)	
Missing, no. (%)	57 (0.1)	254 (0.1)		13 (0.1)	50 (0.1)		44 (0.1)	204 (0.1)	
**Total cholesterol**^e^, **mean ± SD**	200.6 ± 37.6	202.0 ± 37.6	< 0.001	192.3 ± 36.7	194.4 ± 35.8	< 0.001	203.0 ± 37.5	204.2 ± 37.8	< 0.001
**Hemoglobin**^e^, **mean ± SD**	13.2 ± 1.3	13.2 ± 1.4	< 0.001	14.4 ± 1.3	14.6 ± 1.2	< 0.001	12..8 ± 1.1	12.8 ± 1.1	0.053
**Urine occult blood**^**e**^			0.115			0.015			0.018
Negative, no. (%)	48,009 (86.0)	239,853 (86.0)		11,591 (92.8)	58,415 (93.6)		36,418 (84.1)	181,438 (83.8)	
± , no. (%)	1792 (3.2)	9219 (3.3)		223 (1.8)	1069 (1.7)		1569 (3.6)	8150 (3.8)	
Positive, no. (%)	5762 (10.3)	28,557 (10.2)		609 (4.9)	2664 (4.3)		5153 (11.9)	25,893 (12.0)	
Missing, no. (%)	236 (0.4)	1366 (0.5)		63 (0.5)	282 (0.4)		173 (0.4)	1084 (0.5)	
**Urine protein**^**e**^			<0 .001			0.126			< 0.001
Negative, no. (%)	53,871 (96.5)	268,276 (96.2)		11,987 (96.0)	59,842 (95.8)		41,884 (96.7)	208,434 (96.2)	
± , no. (%)	708 (1.3)	3782 (1.4)		164 (1.3)	988 (1.6)		544 (1.3)	2794 (1.3)	
Positive, no. (%)	994 (1.8)	5570 (2.0)		273 (2.2)	1321 (2.1)		721 (1.7)	4249 (2.0)	
Missing, no. (%)	226 (0.4)	1367 (0.5)		62 (0.5)	279 (0.5)		164 (0.4)	1088 (0.5)	
**AST**^c,e^, **mean ± SD**	25.5 ± 14.0	25.4 ± 14.2	0.2	29.1 ± 19.4	28.2 ± 17.8	<0.001	24.5 ± 11.8	24.6 ± 12.9	0.017
**ALT**^c,e^, **mean ± SD**	22.4 ± 15.5	22.7 ± 16.5	<0.001	26.9 ± 18.5	26.9 ± 20.2	0.959	21.1 ± 14.2	21.5 ± 15.1	<0.001
**Gamma-GTP**^c,e^, **mean ± SD**	27.8 ± 41.2	27.4 ± 36.9	0.051	49.5 ± 72.7	45.8 ± 59.8	<0.001	21.5 ± 22.0	22.1 ± 24.5	<0.001
**Smoking status**^**e**^			<0.001			<0.001			0.049
Non-smoker, no. (%)	46,816 (83.9)	234,352 (84.0)		6112 (48.9)	31,352 (50.2)		40,704 (94.0)	203,000 (93.7)	
Ex-smoker, no. (%)	2090 (3.8)	10,865 (3.9)		1806 (14.5)	9217 (14.8)		284 (0.7)	1648 (0.8)	
Current smoker, no. (%)	4726 (8.5)	22,413 (8.0)		3903 (31.3)	18,327 (29.4)		823 (1.9)	4086 (1.9)	
Missing, no. (%)	2167 (3.9)	11,365 (4.1)		665 (5.3)	3534 (5.7)		1502 (3.5)	7831 (3.6)	
**Frequency of drinking alcohol**^**e**^			0.003			<0.001			0.152
< 1 day/week, no. (%)	46,955 (84.2)	234,284 (84.0)		7231 (57.9)	36,056 (57.7)		39,724 (91.7)	198,228 (91.5)	
1–2 days/week, no. (%)	4415 (7.9)	23,025 (8.3)		2466 (19.7)	13,345 (21.4)		1949 (4.5)	9680 (4.5)	
3–7 days/week, no. (%)	3232 (5.8)	15,452 (5.5)		2606 (20.9)	12,030 (19.3)		626 (1.5)	3422 (1.6)	
Missing, no. (%)	1197 (2.2)	6234 (2.2)		183 (1.5)	999 (1.6)		1014 (2.3)	5235 (2.4)	
**Frequency of exercise**^**e**^			<0.001			<0.001			<0.001
< 1 days/week, no. (%)	32,621 (58.5)	153,528 (55.0)		6602 (52.9)	28,797 (46.1)		26,019 (60.1)	124,731 (57.6)	
1–2 days/week, no. (%)	10,611 (19.0)	57,095 (20.5)		2814 (22.5)	16,012 (25.7)		7797 (18.0)	41,083 (19.0)	
Missing, no. (%)	1489 (2.7)	7741 (2.8)		325 (2.6)	1510 (2.4)		1164 (2.7)	6231 (2.9)	
**The number of healthcare visits, mean ± SD** ^d^	110.6 ± 114.4	80.6 ± 88.8	<0.001	142.0 ± 146.5	100 ± 109.4	< 0.001	101.6 ± 101.6	75.0 ± 81.1	< 0.001

^a^Not all 334,794 subjects (total patients: 55,799, total control: 278,995) had information for all listed measures. Specifically, body mass index (BMI) was available for 334,667 subjects; blood pressure was available for 334,674 subjects; fasting blood glucose was available for 334,483 subjects; total cholesterol was available for 334,357 subjects; hemoglobin was available for 334,450 subjects; urine occult blood was available for 333,192 subjects; urine protein was available for 333,201 subjects; AST was available for 334,411 subjects; ALT was available for 334,416 subjects; gamma-GTP was available for 334,489 subjects; smoking status was available for 321,262 subjects; frequency of drinking alcohol was available for 327,363 subjects; and frequency of exercise was available for 325,564 subjects.

^b^Aromatase inhibitors and aromatase deprivation therapy were available only for women and men, respectively.

^c^*DMPA* depot-medroxyprogesterone acetate, *TCA* tricyclic antidepressant, *BMI* body mass index, *SBP* systolic blood pressure, *DBP* diastolic blood pressure, *AST* aspartate transaminase, *ALT* alanine transaminase, *GTP* glutamyltransferase, *CCI* Charlson comorbidity index.

^d^The prescriptions of the medications and the number of healthcare visits were measured during the observation period.

^e^Bio-clinical laboratory results, demographic information and history of smoking and drinking alcohol were measured at the baseline.

^f^The Charlson comorbidity index (CCI) was calculated based on claims collected for two years before the baseline exam.

The association between the use of antidepressant drugs and the risk of subsequent low bone density is shown in Table 2. The odds ratio (OR) was 1.44 (95% CI 1.38, 1.50) for former users and 1.80 (95% CI 1.69, 1.91) for recent users. Use of SRIs remained an independent predictor of subsequent low bone density after adjusting for potential confounders (glucocorticoids, aromatase inhibitors, androgen deprivation therapy, GnRH agonists, anticonvulsants, thiazolidinediones, DMPA, antipsychotics, benzodiazepine, TCA, Estrogen or Estrogen and progesterone combination, household income, CCI, BMI, systolic and diastolic blood pressures, fasting blood glucose level, total cholesterol level, hemoglobin level, urine occult blood, urine protein, AST, ALT, smoking status, frequency of drinking alcohol, and frequency of exercise). After adjusting for those potential confounders, former use of SRIs was associated with a 1.07-fold higher risk of low bone density than was found among non-users (OR 1.07, 95% 1.03, 1.12), and the risk for recent users was even higher (OR 1.44, 95% CI 1.35, 1.53). The results of the multivariable models showed a similar trend in men and women. In men, the OR was 1.10 (95% CI 1.01, 1.20) for former users and 1.35 (95% CI 1.20, 1.53) for recent users. Among women, former users had a 1.07-fold higher risk of low bone density than non-users (OR 1.07, 95% CI 1.02–1.12), and the risk of low bone density in women increased by 1.38-fold in recent users compared with non-users (OR 1.38, 95% CI 1.28, 1.48). In the sensitivity analysis to examine confounding by frequent healthcare utilization, while the association was not significant for either sex in former users (OR 1.04, 95% CI 0.99 -1.09, Men: OR 1.07, 95% CI 0.98–1.17, Women: OR 1.02, 95% CI 0.97–1.08), the association between the use of SRIs and the risk of bone loss remained statistically significant in recent users (OR 1.34, 95% CI 1.25–1.42, Men: OR 1.29 95% CI 1.14–1.46, Women: OR 1.22 95% CI 1.13–1.31). In the recent SRI users, the mean period between the initiation of SRI treatment and the diagnosis of low bone density was 1.8 years (SD = 2.2 years).

**Table 2:** Use of Serotonin reuptake inhibitors and the risk of low bone density.

	Crude odds ratio (95% CI)			Adjusted odds ratio (95% CI)^a^		
	Total	Men	Women	Total	Men	Women
Non-users	1.00 (ref)	1.00 (ref)	1.00 (ref)	1.00(ref)	1.00 (ref)	1.00 (ref)
Former users	1.44 (1.38–1.50)	1.55 (1.43–1.69)	1.41 (1.35–1.48)	1.07 (1.03–1.12)	1.10 (1.01–1.20)	1.07 (1.02–1.12)
Recent users	1.80 (1.69–1.91)	2.13 (1.90–2.39)	1.69 (1.57–1.81)	1.44 (1.35–1.53)	1.35 (1.20–1.53)	1.38 (1.28–1.48)

^a^Adjusted for: glucocorticoids, aromatase inhibitors, androgen deprivation therapy, GnRH agonists, anticonvulsants, thiazolidinediones, DMPA, antipsychotics, benzodiazepine, TCA, Estrogen or Estrogen and progesterone combination, household income, CCI, BMI, systolic and diastolic blood pressures, fasting blood glucose level, total cholesterol level, hemoglobin level, urine occult blood, urine protein, AST, ALT, smoking status, frequency of drinking alcohol, and frequency of exercise.

## Discussion

This real-world nationwide, longitudinal cohort study investigated the association between the use of SRIs and subsequent low bone density, stratifying participants by their time-dependent use of the medications and their sex. Those exposed to SRIs had an increased risk of newly diagnosed low bone density (osteoporosis or osteopenia) compared with those who did not receive those medications. After adjusting for multiple confounding factors, the association remained significant, suggesting an independent association between the use of SRIs and the risk of osteoporosis. that the use of SRIs independently altered the risk of osteoporosis. After adjusting for confounding factors, the risk of subsequent low bone density was 1.44-fold higher in recent users and 1.07-fold higher in former users compared with the risk in non-users. Given the high prevalence of SRI use, these findings could have significant clinical implications.

To the best of our knowledge, this is the largest nationwide cohort study (74,916 men and 259,878 women) to examine the association between the use of SRIs and the risk of bone loss. Our finding that the risk of low bone density was higher in people who took SRIs in a population-based Korean cohort supports the hypothesis that the use of SRIs is associated with an increased risk of bone loss. Although little clinical attention has been paid to the potential adverse effects of SRIs on bones, several reports have linked the use of SRIs to an increased risk of osteoporosis^6–8,24^ (other studies did not show any link between SRI use and bone health^10,21^). In addition, we found significant associations between SRIs and low bone density in both men and women, whereas mixed and inconsistent results were shown in previous studies with smaller sample sizes^8,22^. A cross-sectional study of 5995 men reported that SSRI users had 3.7% lower BMD in their hips and 5.9% lower BMD in their lumber spines^8^, but another study of 141 men did not find any significant difference in BMD among users and non-users of antidepressants^22^. According to a meta-analysis of 11 observational studies, women taking SSRIs had significantly lower bone mass in their lumbar spine than non-users, but that paper failed to make a comprehensive analysis in men because of an insufficient number of subjects in previous studies^24^. Our findings are based on a large, well-defined population and demonstrate that the use of SRIs is associated with an increased risk of subsequent bone loss in both sexes. Therefore, people who taking SRIs should be monitored for their bone health.

The mechanism underlying the relationship between bone loss and SRI use remains poorly understood. Although we did not examine the causal mechanism in this study, the role of serotonin receptors in bone is a potential molecular mechanism. Several studies have shown the presence of serotonin receptors and transporters in osteoblasts and osteoclasts, which are the key elements in the bone remodeling cycle, and suggested possible adverse effects, such as reduced osteoblast proliferation, from blockading serotonin reuptake during bone remodeling^25–29^. An animal study proposed that SSRIs could reduce BMD by altering osteoclast differentiation and increasing sympathetic tone^29^, and that possibility warrants future research.

In our multivariable models, recent and former SRI users were associated with 1.44-fold and 1.07-fold increased risk of subsequent low bone density, respectively, compared with non-users, indicating that the risk in recent users is higher than that in former users. In addition, the sensitivity analysis examining confounding by frequent healthcare utilization revealed that while the association was no longer significant in former SRI users, a statistically significant association between the use of SRIs and the risk of bone loss was observed in recent users. These results suggest that recent users are at higher risk for bone loss than former users and that discontinuing SRIs might reverse the bone loss related to SRI use. In line with that finding, several studies have reported that although current use of antidepressants is associated with an increased risk of bone fractures compared with non-users, no significant association was found in former users^13,17,30^. Furthermore, one cohort study that investigated the association between the time since the last use of an SSRI and bone fractures found that the risk of osteoporotic fractures declined rapidly after discontinuing SRI use^31^. Further research is needed to better understand the reversible or irreversible effects of SRIs on bone health and their underlying mechanisms.

Given the high prevalence of depressive disorders and SRI use, the association between SRI exposure and risk of bone loss could lead to additional public health burdens. Therefore, it is important to pay attention to the issue of the adverse effects of SRIs on bone, especially in individuals who recently took or are currently taking them.

The major strength of our study is the use of a nationwide, longitudinal database, which ensured minimal selection bias. The database included the serial results of healthcare utilization without any losses, and the data were collected uniformly among cases and controls. Furthermore, because the NHIS database includes prescription history and lifestyle variables, we were able to adjust for multiple potential confounding variables, including alcohol use, smoking, and exercise status and the use of other medicines known to induce low BMD.

Nevertheless, this study had several limitations. First, the newly diagnosed cases of osteoporosis and osteopenia in this study could be potentially susceptible to errors of misclassification. To increase the diagnostic accuracy, we applied a strict operational diagnosis of low bone density with appropriate ICD-10 codes and prescriptions for an osteoporosis-related medication. This operational definition is reliable, because the NHIS allows the prescription of osteoporosis-related medications only if the T score derived from DEXA is lower than -1. However, since the baseline bone mineral density of subjects at inclusion and the extent of bone loss in the form of T or Z scores during the observation period were not available the database, it is possible that a low bone mass at the index date resulted from a baseline bone mass at the lower end of the normal range, and not necessarily due to increased bone loss. Second, because the present database of insurance claims does not provide information about patient adherence to an SRI regime, the possibility that some individuals with poor drug compliance biased the results cannot be completely excluded. Third, frequent healthcare utilization may lead to potential detection bias of low bone density, although our sensitivity analysis revealed that the main results remained statistically significant in the recent SRI users. Lastly, we could not consider depression itself as a covariate, which may be a risk factor for osteoporosis^8,32,33^. Although the causal link between depression and bone loss is disputed, the physiological alterations associated with depression, such as dysregulation of hypothalamus–pituitary–adrenal axis^34^ and sympathetic systems and inflammatory immune responses including enhanced pro-inflammatory cytokines^35,36^, might affect bone mass. In addition, lifestyle patterns in people with depression, such as low physical activities and unhealthy diet, might contribute to osteoporosis^37^. Further research is needed to clarify the associations between depression and bone loss. Additionally, other confounders that could affect osteoporosis, such as serum vitamin D deficiency^38^, and menopause status^39^, were not considered.

In conclusion, this nationwide population-based study showed that the use of SRIs is associated with an increased risk of subsequent bone loss. In particular, recent users were associated with a higher risk of low bone density than former users. These findings provide reliable, population-level evidence for the risk of bone loss associated with SRI exposure, and highlight the importance of carefully monitoring bone loss in individuals taking SRIs.

## Method

### Study cohort

We used the National Health Insurance Sharing Service-National Health Screening (NHIS-HEALS) cohort, a representative sample cohort database from health screening participants that has been made publicly available and contains both comprehensive health screening data and long-term health outcomes. The cohort includes 514,866 participants, a 10% random sample of all health screening participants aged 40 to 79 years in 2002, and those people were repeatedly followed up until 2015^40^. The cohort provides access to variables such as sociodemographic information, specific health problems (e.g., smoking, alcohol use, medical history, and family history), bio-clinical laboratory results, diagnoses in the form of International Classification of Disease 10th revision [ICD-10] codes, drug codes, date and daily dosage of prescriptions, and date and cause of death.

Within the NHIS-HEALS database, participants who had received a medical check-up between Jan 2004 and Dec 2006 were selected. The date of the medical check-up between 2004 and 2006 was taken as the baseline. Subjects with a preexisting diagnosis related to bone loss (osteoporosis or osteopenia) or exposure to SRIs at least for 2 years before the baseline were excluded (washout period). The date (before 2015) on which participants received both a diagnosis of low bone density and a prescription for an osteoporosis-related medication was regarded as the index date. The period between the baseline and the index date is the observation period. Using a 1:5 matching ratio of cases to controls, controls without any diagnosis related to bone loss were randomly selected based on age, sex, date of medical check-up, and length of the observation period for each case in the group with low bone mass.

### Outcome

The primary outcome was a new diagnosis of low bone density (osteoporosis or osteopenia) after exposure to SRIs. To increase the accuracy of diagnosis, the operational definition of low bone density was defined strictly as meeting all of the following requirements: first, a diagnostic code corresponding to osteoporosis or osteopenia (ICD-10 codes: M80, M81, M82, M85.8) during the observational period, second, prescription of osteoporosis-related medication on the same date as diagnosis and third, no diagnosis of osteoporosis or osteopenia or the prescription of SRIs during the washout period. In Korea, prescribing osteoporosis-related medication is allowed only if the T score derived from DEXA is lower than -1, a definition of low bone density that strongly supports the diagnosis of osteoporosis or osteopenia. The qualifying osteoporosis-related medications were vitamin D and its analogues (alfacalcidol, calcitriol, calcifediol), calcium (calcium gluconate, calcium carbonate, calcium citrate, ossopan substance, oyster shell powder), bisphosphonate (etidronic acid, pamidronic acid, alendronic acid, ibandronic acid, risedronic acid, zoledronic acid), calcitonin (salmon synthetic calcitonin, salcatonin, elcatonin), selective estrogen receptor modulators (raloxifene, bazedoxifene), and combination agents (cholecalciferol + ibandronic acid, cholecalciferol + alendronic acid, cholecalciferol + risedronic acid).

### Exposure

Because the database includes drug codes and prescription dates, participants who were prescribed SRIs during the observation period were considered to be exposed to SRIs. Furthermore, participants were classified into three time-dependent user groups as follows. Participants who did not receive a prescription for SRIs from the baseline to the index date were considered *non-users*. Participants exposed to an SRI after the baseline who stopped taking the medication at least 6 months before the index date were considered *former users*. Participants who were prescribed of SRIs during the last 6 months of the observation period were considered *recent users*. The SRIs considered in this study were escitalopram, citalopram, sertraline, fluoxetine, fluvoxamine, paroxetine, trazodone, venlafaxine, desvenlafaxine, duloxetine, and milnacipran. The period between the initiation of SRI treatment and the diagnosis of low bone density in the recent SRI users was assessed.

### Covariates

The following covariates were measured during the baseline exam: blood pressure (systolic, diastolic)^41^, body mass index (BMI), fasting blood glucose, total cholesterol, urine occult blood, urine protein, aspartate transaminase (AST), alanine transaminase (ALT), and Gamma-Glutamyltransferase (GTP)^42^. The analyses were also adjusted for demographic information (age, sex, household income)^42–44^, smoking status (non-smoker, ex-smoker, current smoker), the number of days of alcohol consumption per week, and the number of days of exercise per week^42^, which were collected through self-reported questionnaires and administration data at baseline. To control for the possible effects of other medications prescribed during the observation period, we made adjustments for several medications based on previous studies of low bone mass^42,45^: glucocorticoids, aromatase inhibitors, androgen deprivation therapy, gonadotropin-releasing hormone (GnRH) agonists, anticonvulsants, thiazolidinediones, depot-medroxyprogesterone acetate (DMPA), antipsychotics, benzodiazepine, tricyclic antidepressant (TCA : clomipramine, amitriptyline, amoxapine, imipramine, nortriptyline, doxepin), estrogen, and estrogen progesterone combination therapy. To control for medical comorbidities, the Charlson Comorbidity Index (CCI)^42^ was calculated based on claims collected for two years before the baseline exam. In addition, the number of healthcare visits during the observation period was measured.

### Statistical analysis

To compare the baseline characteristics of the study sample, χ^2^ tests and t-tests were used for categorical variables and continuous data, respectively. A nested case–control analysis, commonly used in cohort studies to eliminate immortal-time bias, was used to determine the association between the use of SRIs and the incidence of a new diagnosis of low bone density. In a nested case–control study design, for each case, a specified number of controls who were free of disease on the diagnosis date of their corresponding cases were matched^46^. A conditional logistic regression was used to estimate the odds ratios (ORs) and corresponding 95% confidence intervals (CIs). We included the following covariates in the multivariable models: medication (glucocorticoids, aromatase inhibitors, androgen deprivation therapy, GnRH agonists, anticonvulsants, thiazolidinediones, DMPA, antipsychotics, benzodiazepine, TCA, estrogen, and estrogen progesterone combination therapy), demographic information (household income), CCI, physiological measures (systolic blood pressure, diastolic blood pressure, BMI, fasting blood glucose, total cholesterol, urine occult blood, urine protein, AST, ALT, and Gamma- GTP), and lifestyle habits (smoking status, frequency of drinking alcohol, and frequency of exercise). Furthermore, since subjects with poorer health conditions may be more likely to show a higher frequency of healthcare utilization, thus having higher chances of detecting osteoporosis or the prescription of SRIs, we conducted a sensitivity analysis including the number of healthcare visits as an additional covariate. The SAS Enterprise Guide was used for all statistical analyses, and *p*-values less than 0.05 were considered statistically significant.

### Ethics

This study was approved by the Institutional Review Board of Yonsei University Severance Hospital (IRB No. 4-2019-0017) and was conducted in accordance with the principles of the Declaration of Helsinki.

## Data Availability

The data that support the findings of this study are available from the National Health Insurance Service in South Korea. Restrictions apply to the availability of these data, which were used under license for this study, and so are not publicly available. Data are available from the authors upon reasonable request and with permission of the National Health Insurance Service.
